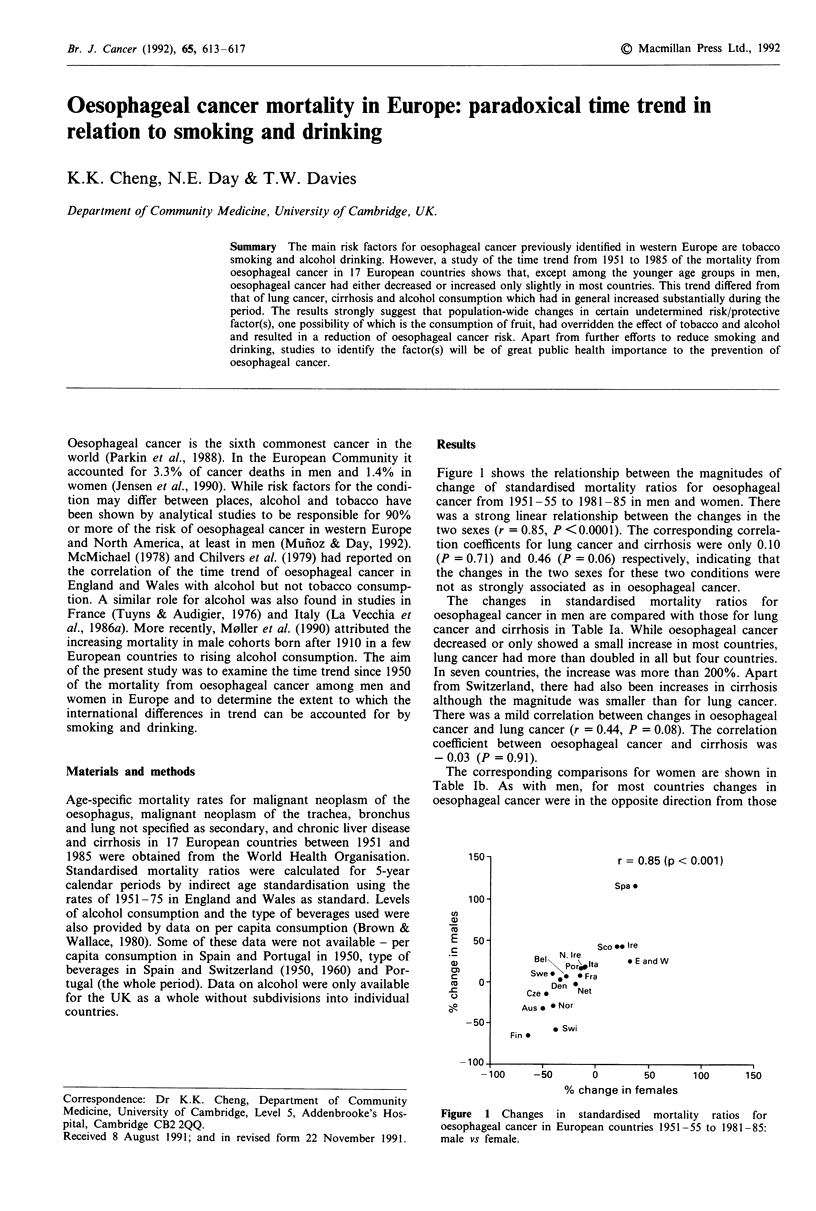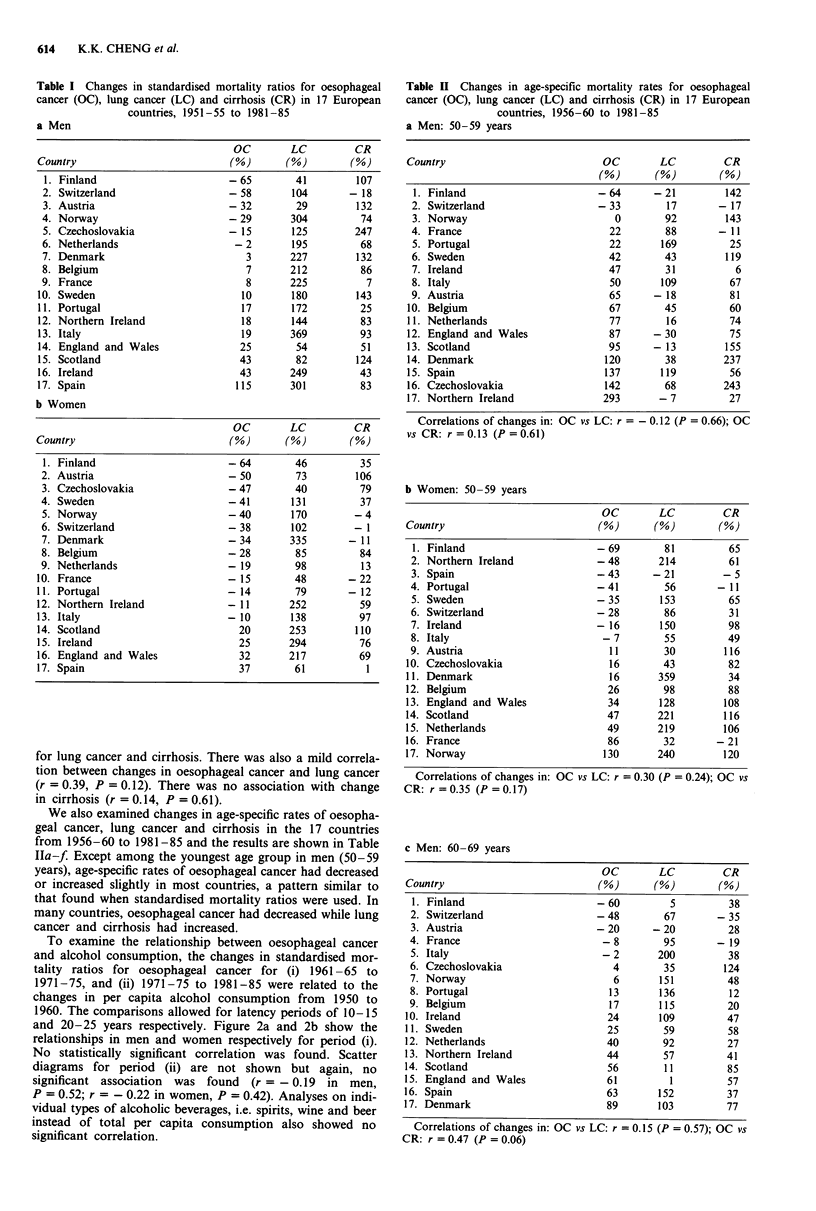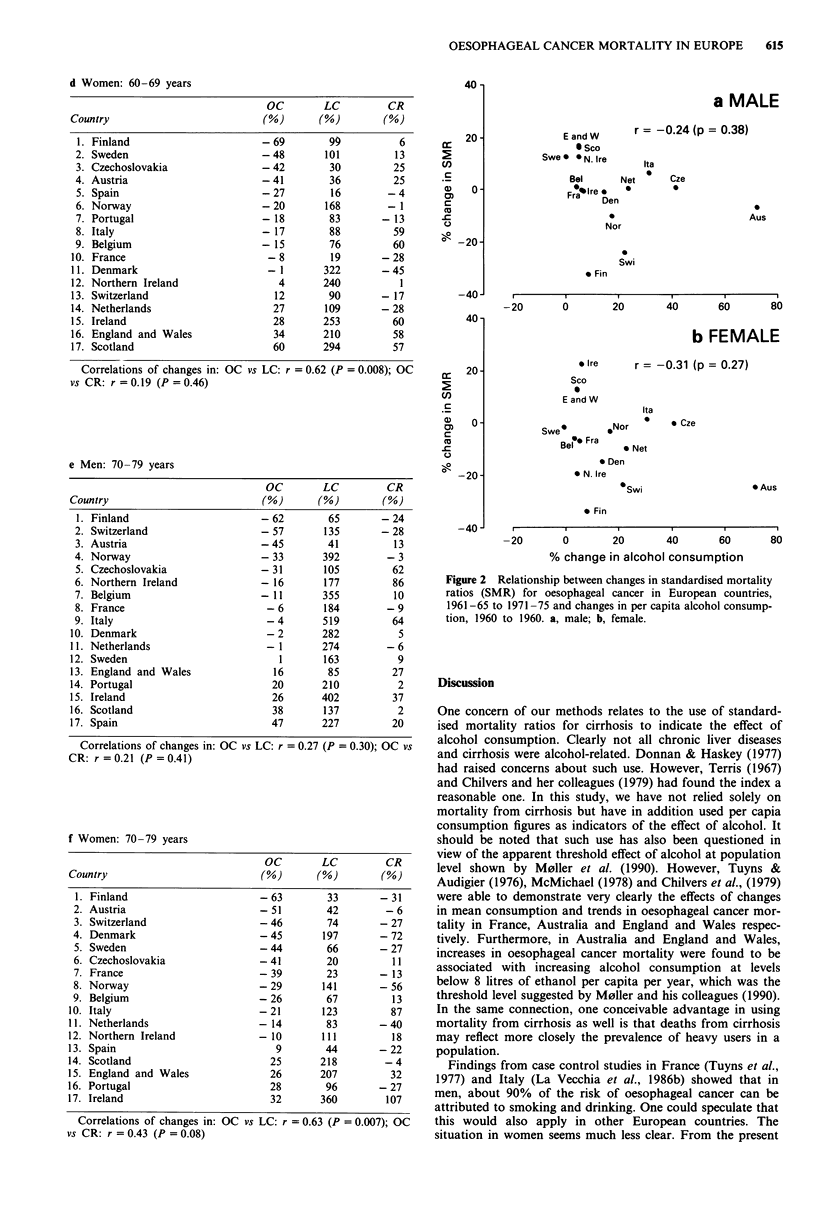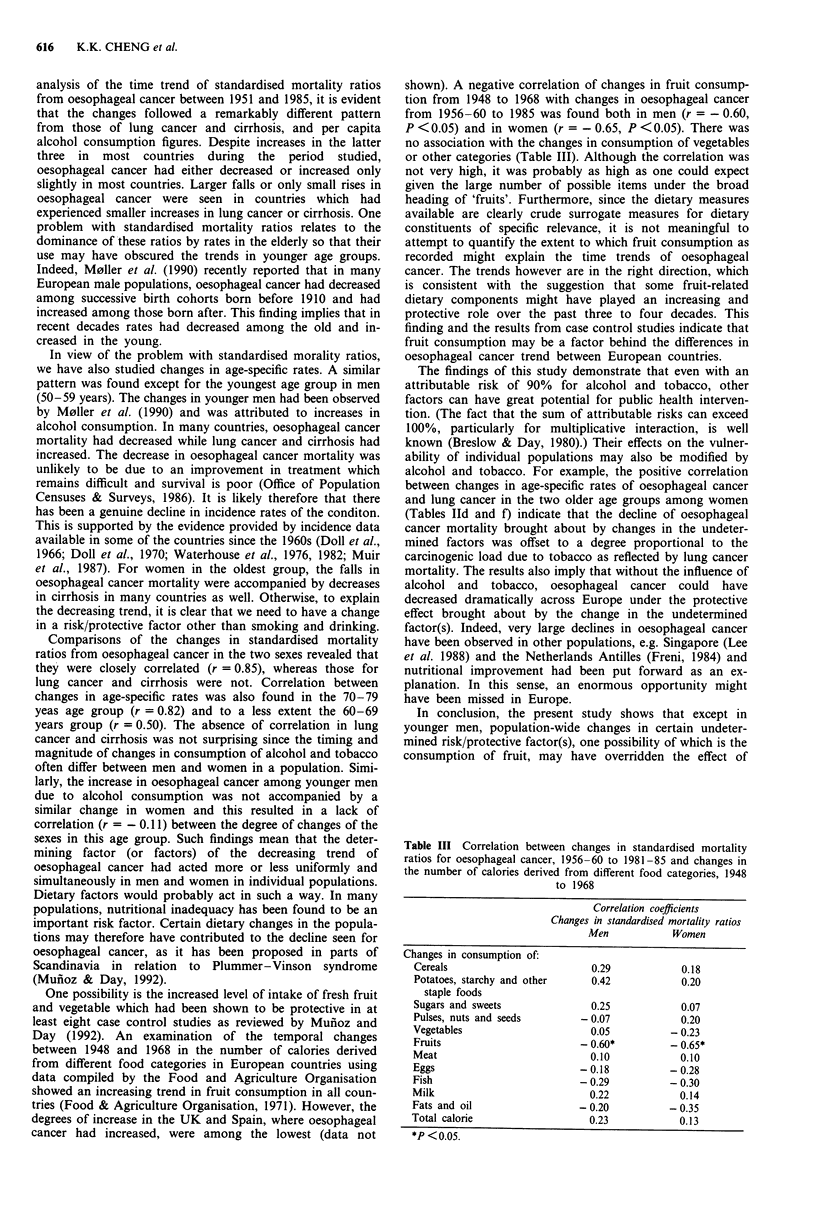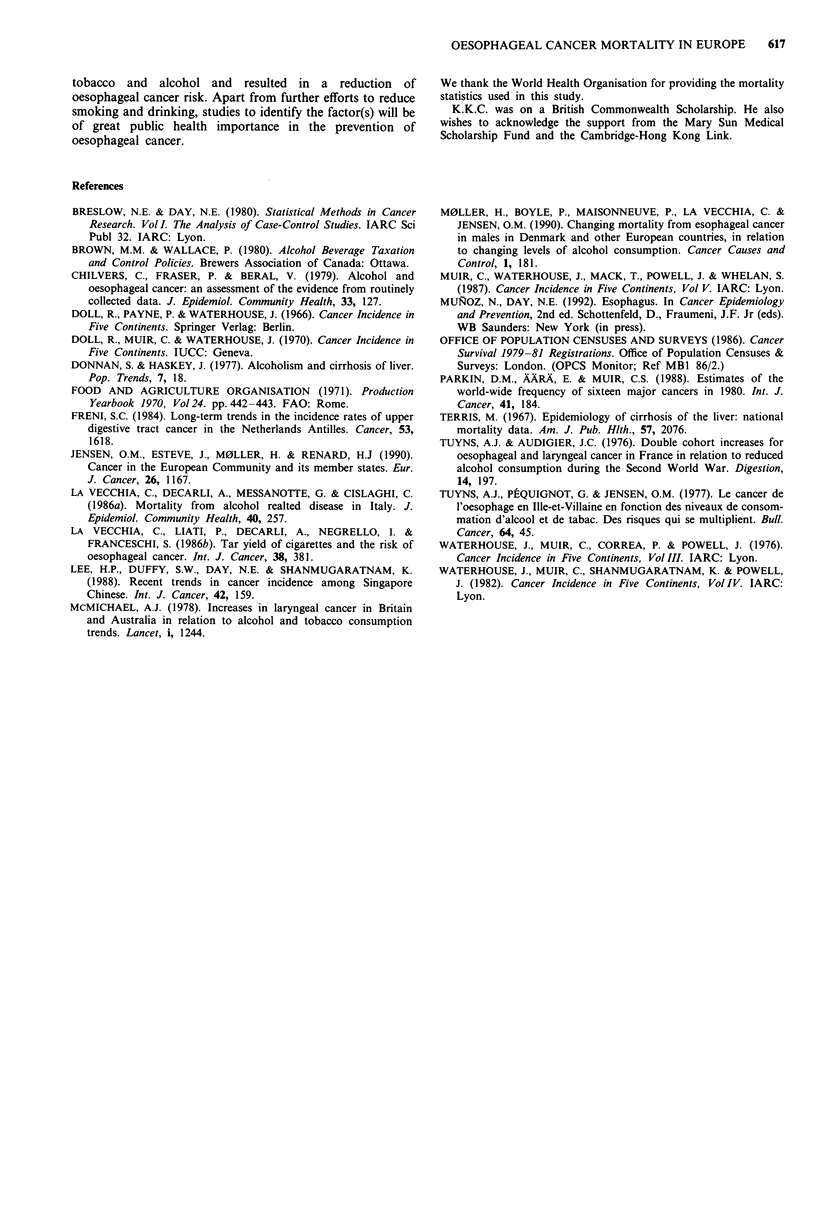# Oesophageal cancer mortality in Europe: paradoxical time trend in relation to smoking and drinking.

**DOI:** 10.1038/bjc.1992.124

**Published:** 1992-04

**Authors:** K. K. Cheng, N. E. Day, T. W. Davies

**Affiliations:** Department of Community Medicine, University of Cambridge, UK.

## Abstract

The main risk factors for oesophageal cancer previously identified in western Europe are tobacco smoking and alcohol drinking. However, a study of the time trend from 1951 to 1985 of the mortality from oesophageal cancer in 17 European countries shows that, except among the younger age groups in men, oesophageal cancer had either decreased or increased only slightly in most countries. This trend differed from that of lung cancer, cirrhosis and alcohol consumption which had in general increased substantially during the period. The results strongly suggest that population-wide changes in certain undetermined risk/protective factor(s), one possibility of which is the consumption of fruit, had overridden the effect of tobacco and alcohol and resulted in a reduction of oesophageal cancer risk. Apart from further efforts to reduce smoking and drinking, studies to identify the factor(s) will be of great public health importance to the prevention of oesophageal cancer.


© Macmillan Press Ltd., 1992

### Oesophageal cancer mortality in Europe: paradoxical time trend in relation to smoking and drinking

K.K. Cheng, N.E. Day & T.W. Davies

*Department of Community Medicine, University of Cambridge, UK.*

**Summary** The main risk factors for oesophageal cancer previously identified in western Europe are tobacco smoking and alcohol drinking. However, a study of the time trend from 1951 to 1985 of the mortality from oesophageal cancer in 17 European countries shows that, except among the younger age groups in men, oesophageal cancer had either decreased or increased only slightly in most countries. This trend differed from that of lung cancer, cirrhosis and alcohol consumption which had in general increased substantially during the period. The results strongly suggest that population-wide changes in certain undetermined risk/protective factor(s), one possibility of which is the consumption of fruit, had overridden the effect of tobacco and alcohol and resulted in a reduction of oesophageal cancer risk. Apart from further efforts to reduce smoking and drinking, studies to identify the factor(s) will be of great public health importance to the prevention of oesophageal cancer.

Oesophageal cancer is the sixth commonest cancer in the world (Parkin *et al.*, 1988). In the European Community it accounted for 3.3% of cancer deaths in men and 1.4% in women (Jensen *et al.*, 1990). While risk factors for the condition may differ between places, alcohol and tobacco have been shown by analytical studies to be responsible for 90% or more of the risk of oesophageal cancer in western Europe and North America, at least in men (Muñoz & Day, 1992). McMichael (1978) and Chilvers *et al.* (1979) had reported on the correlation of the time trend of oesophageal cancer in England and Wales with alcohol but not tobacco consumption. A similar role for alcohol was also found in studies in France (Tuyns & Audigier, 1976) and Italy (La Vecchia *et al.*, 1986*a*). More recently, Møller *et al.* (1990) attributed the increasing mortality in male cohorts born after 1910 in a few European countries to rising alcohol consumption. The aim of the present study was to examine the time trend since 1950 of the mortality from oesophageal cancer among men and women in Europe and to determine the extent to which the international differences in trend can be accounted for by smoking and drinking.

### Materials and methods

Age-specific mortality rates for malignant neoplasm of the oesophagus, malignant neoplasm of the trachea, bronchus and lung not specified as secondary, and chronic liver disease and cirrhosis in 17 European countries between 1951 and 1985 were obtained from the World Health Organisation. Standardised mortality ratios were calculated for 5-year calendar periods by indirect age standardisation using the rates of 1951–75 in England and Wales as standard. Levels of alcohol consumption and the type of beverages used were also provided by data on per capita consumption (Brown & Wallace, 1980). Some of these data were not available – per capita consumption in Spain and Portugal in 1950, type of beverages in Spain and Switzerland (1950, 1960) and Portugal (the whole period). Data on alcohol were only available for the UK as a whole without subdivisions into individual countries.

Correspondence: Dr K.K. Cheng, Department of Community Medicine, University of Cambridge, Level 5, Addenbrooke's Hospital, Cambridge CB2 2QQ.
Received 8 August 1991; and in revised form 22 November 1991.

### Results

Figure 1 shows the relationship between the magnitudes of change of standardised mortality ratios for oesophageal cancer from 1951–55 to 1981–85 in men and women. There was a strong linear relationship between the changes in the two sexes ($r = 0.85$, $P < 0.0001$). The corresponding correlation coefficents for lung cancer and cirrhosis were only 0.10 ($P = 0.71$) and 0.46 ($P = 0.06$) respectively, indicating that the changes in the two sexes for these two conditions were not as strongly associated as in oesophageal cancer.

The changes in standardised mortality ratios for oesophageal cancer in men are compared with those for lung cancer and cirrhosis in Table Ia. While oesophageal cancer decreased or only showed a small increase in most countries, lung cancer had more than doubled in all but four countries. In seven countries, the increase was more than 200%. Apart from Switzerland, there had also been increases in cirrhosis although the magnitude was smaller than for lung cancer. There was a mild correlation between changes in oesophageal cancer and lung cancer ($r = 0.44$, $P = 0.08$). The correlation coefficient between oesophageal cancer and cirrhosis was $-0.03$ ($P = 0.91$).

The corresponding comparisons for women are shown in Table Ib. As with men, for most countries changes in oesophageal cancer were in the opposite direction from those

**Figure 1** Changes in standardised mortality ratios for oesophageal cancer in European countries 1951–55 to 1981–85: male *vs* female.

**Table I** Changes in standardised mortality ratios for oesophageal cancer (OC), lung cancer (LC) and cirrhosis (CR) in 17 European countries, 1951–55 to 1981–85

**a** Men

| Country | OC (%) | LC (%) | CR (%) |
|---|---|---|---|
| 1. Finland | – 65 | 41 | 107 |
| 2. Switzerland | – 58 | 104 | – 18 |
| 3. Austria | – 32 | 29 | 132 |
| 4. Norway | – 29 | 304 | 74 |
| 5. Czechoslovakia | – 15 | 125 | 247 |
| 6. Netherlands | – 2 | 195 | 68 |
| 7. Denmark | 3 | 227 | 132 |
| 8. Belgium | 7 | 212 | 86 |
| 9. France | 8 | 225 | 7 |
| 10. Sweden | 10 | 180 | 143 |
| 11. Portugal | 17 | 172 | 25 |
| 12. Northern Ireland | 18 | 144 | 83 |
| 13. Italy | 19 | 369 | 93 |
| 14. England and Wales | 25 | 54 | 51 |
| 15. Scotland | 43 | 82 | 124 |
| 16. Ireland | 43 | 249 | 43 |
| 17. Spain | 115 | 301 | 83 |

**b** Women

| Country | OC (%) | LC (%) | CR (%) |
|---|---|---|---|
| 1. Finland | – 64 | 46 | 35 |
| 2. Austria | – 50 | 73 | 106 |
| 3. Czechoslovakia | – 47 | 40 | 79 |
| 4. Sweden | – 41 | 131 | 37 |
| 5. Norway | – 40 | 170 | – 4 |
| 6. Switzerland | – 38 | 102 | – 1 |
| 7. Denmark | – 34 | 335 | – 11 |
| 8. Belgium | – 28 | 85 | 84 |
| 9. Netherlands | – 19 | 98 | 13 |
| 10. France | – 15 | 48 | – 22 |
| 11. Portugal | – 14 | 79 | – 12 |
| 12. Northern Ireland | – 11 | 252 | 59 |
| 13. Italy | – 10 | 138 | 97 |
| 14. Scotland | 20 | 253 | 110 |
| 15. Ireland | 25 | 294 | 76 |
| 16. England and Wales | 32 | 217 | 69 |
| 17. Spain | 37 | 61 | 1 |

for lung cancer and cirrhosis. There was also a mild correlation between changes in oesophageal cancer and lung cancer ($r = 0.39$, $P = 0.12$). There was no association with change in cirrhosis ($r = 0.14$, $P = 0.61$).

We also examined changes in age-specific rates of oesophageal cancer, lung cancer and cirrhosis in the 17 countries from 1956–60 to 1981–85 and the results are shown in Table II*a–f*. Except among the youngest age group in men (50–59 years), age-specific rates of oesophageal cancer had decreased or increased slightly in most countries, a pattern similar to that found when standardised mortality ratios were used. In many countries, oesophageal cancer had decreased while lung cancer and cirrhosis had increased.

To examine the relationship between oesophageal cancer and alcohol consumption, the changes in standardised mortality ratios for oesophageal cancer for (i) 1961–65 to 1971–75, and (ii) 1971–75 to 1981–85 were related to the changes in per capita alcohol consumption from 1950 to 1960. The comparisons allowed for latency periods of 10–15 and 20–25 years respectively. Figure 2a and 2b show the relationships in men and women respectively for period (i). No statistically significant correlation was found. Scatter diagrams for period (ii) are not shown but again, no significant association was found ($r = -0.19$ in men, $P = 0.52$; $r = -0.22$ in women, $P = 0.42$). Analyses on individual types of alcoholic beverages, i.e. spirits, wine and beer instead of total per capita consumption also showed no significant correlation.

**Table II** Changes in age-specific mortality rates for oesophageal cancer (OC), lung cancer (LC) and cirrhosis (CR) in 17 European countries, 1956–60 to 1981–85

**a** Men: 50–59 years

| Country | OC (%) | LC (%) | CR (%) |
|---|---|---|---|
| 1. Finland | – 64 | – 21 | 142 |
| 2. Switzerland | – 33 | 17 | – 17 |
| 3. Norway | 0 | 92 | 143 |
| 4. France | 22 | 88 | – 11 |
| 5. Portugal | 22 | 169 | 25 |
| 6. Sweden | 42 | 43 | 119 |
| 7. Ireland | 47 | 31 | 6 |
| 8. Italy | 50 | 109 | 67 |
| 9. Austria | 65 | – 18 | 81 |
| 10. Belgium | 67 | 45 | 60 |
| 11. Netherlands | 77 | 16 | 74 |
| 12. England and Wales | 87 | – 30 | 75 |
| 13. Scotland | 95 | – 13 | 155 |
| 14. Denmark | 120 | 38 | 237 |
| 15. Spain | 137 | 119 | 56 |
| 16. Czechoslovakia | 142 | 68 | 243 |
| 17. Northern Ireland | 293 | – 7 | 27 |

Correlations of changes in: OC *vs* LC: $r = -0.12$ ($P = 0.66$); OC *vs* CR: $r = 0.13$ ($P = 0.61$)

**b** Women: 50–59 years

| Country | OC (%) | LC (%) | CR (%) |
|---|---|---|---|
| 1. Finland | – 69 | 81 | 65 |
| 2. Northern Ireland | – 48 | 214 | 61 |
| 3. Spain | – 43 | – 21 | – 5 |
| 4. Portugal | – 41 | 56 | – 11 |
| 5. Sweden | – 35 | 153 | 65 |
| 6. Switzerland | – 28 | 86 | 31 |
| 7. Ireland | – 16 | 150 | 98 |
| 8. Italy | – 7 | 55 | 49 |
| 9. Austria | 11 | 30 | 116 |
| 10. Czechoslovakia | 16 | 43 | 82 |
| 11. Denmark | 16 | 359 | 34 |
| 12. Belgium | 26 | 98 | 88 |
| 13. England and Wales | 34 | 128 | 108 |
| 14. Scotland | 47 | 221 | 116 |
| 15. Netherlands | 49 | 219 | 106 |
| 16. France | 86 | 32 | – 21 |
| 17. Norway | 130 | 240 | 120 |

Correlations of changes in: OC *vs* LC: $r = 0.30$ ($P = 0.24$); OC *vs* CR: $r = 0.35$ ($P = 0.17$)

**c** Men: 60–69 years

| Country | OC (%) | LC (%) | CR (%) |
|---|---|---|---|
| 1. Finland | – 60 | 5 | 38 |
| 2. Switzerland | – 48 | 67 | – 35 |
| 3. Austria | – 20 | – 20 | 28 |
| 4. France | – 8 | 95 | – 19 |
| 5. Italy | – 2 | 200 | 38 |
| 6. Czechoslovakia | 4 | 35 | 124 |
| 7. Norway | 6 | 151 | 48 |
| 8. Portugal | 13 | 136 | 12 |
| 9. Belgium | 17 | 115 | 20 |
| 10. Ireland | 24 | 109 | 47 |
| 11. Sweden | 25 | 59 | 58 |
| 12. Netherlands | 40 | 92 | 27 |
| 13. Northern Ireland | 44 | 57 | 41 |
| 14. Scotland | 56 | 11 | 85 |
| 15. England and Wales | 61 | 1 | 57 |
| 16. Spain | 63 | 152 | 37 |
| 17. Denmark | 89 | 103 | 77 |

Correlations of changes in: OC *vs* LC: $r = 0.15$ ($P = 0.57$); OC *vs* CR: $r = 0.47$ ($P = 0.06$)

**d** Women: 60–69 years

| Country | OC (%) | LC (%) | CR (%) |
|---|---|---|---|
| 1. Finland | −69 | 99 | 6 |
| 2. Sweden | −48 | 101 | 13 |
| 3. Czechoslovakia | −42 | 30 | 25 |
| 4. Austria | −41 | 36 | 25 |
| 5. Spain | −27 | 16 | −4 |
| 6. Norway | −20 | 168 | −1 |
| 7. Portugal | −18 | 83 | −13 |
| 8. Italy | −17 | 88 | 59 |
| 9. Belgium | −15 | 76 | 60 |
| 10. France | −8 | 19 | −28 |
| 11. Denmark | −1 | 322 | −45 |
| 12. Northern Ireland | 4 | 240 | 1 |
| 13. Switzerland | 12 | 90 | −17 |
| 14. Netherlands | 27 | 109 | −28 |
| 15. Ireland | 28 | 253 | 60 |
| 16. England and Wales | 34 | 210 | 58 |
| 17. Scotland | 60 | 294 | 57 |

Correlations of changes in: OC *vs* LC: $r = 0.62$ ($P = 0.008$); OC *vs* CR: $r = 0.19$ ($P = 0.46$)

**e** Men: 70–79 years

| Country | OC (%) | LC (%) | CR (%) |
|---|---|---|---|
| 1. Finland | −62 | 65 | −24 |
| 2. Switzerland | −57 | 135 | −28 |
| 3. Austria | −45 | 41 | 13 |
| 4. Norway | −33 | 392 | −3 |
| 5. Czechoslovakia | −31 | 105 | 62 |
| 6. Northern Ireland | −16 | 177 | 86 |
| 7. Belgium | −11 | 355 | 10 |
| 8. France | −6 | 184 | −9 |
| 9. Italy | −4 | 519 | 64 |
| 10. Denmark | −2 | 282 | 5 |
| 11. Netherlands | −1 | 274 | −6 |
| 12. Sweden | 1 | 163 | 9 |
| 13. England and Wales | 16 | 85 | 27 |
| 14. Portugal | 20 | 210 | 2 |
| 15. Ireland | 26 | 402 | 37 |
| 16. Scotland | 38 | 137 | 2 |
| 17. Spain | 47 | 227 | 20 |

Correlations of changes in: OC *vs* LC: $r = 0.27$ ($P = 0.30$); OC *vs* CR: $r = 0.21$ ($P = 0.41$)

**f** Women: 70–79 years

| Country | OC (%) | LC (%) | CR (%) |
|---|---|---|---|
| 1. Finland | −63 | 33 | −31 |
| 2. Austria | −51 | 42 | −6 |
| 3. Switzerland | −46 | 74 | −27 |
| 4. Denmark | −45 | 197 | −72 |
| 5. Sweden | −44 | 66 | −27 |
| 6. Czechoslovakia | −41 | 20 | 11 |
| 7. France | −39 | 23 | −13 |
| 8. Norway | −29 | 141 | −56 |
| 9. Belgium | −26 | 67 | 13 |
| 10. Italy | −21 | 123 | 87 |
| 11. Netherlands | −14 | 83 | −40 |
| 12. Northern Ireland | −10 | 111 | 18 |
| 13. Spain | 9 | 44 | −22 |
| 14. Scotland | 25 | 218 | −4 |
| 15. England and Wales | 26 | 207 | 32 |
| 16. Portugal | 28 | 96 | −27 |
| 17. Ireland | 32 | 360 | 107 |

Correlations of changes in: OC *vs* LC: $r = 0.63$ ($P = 0.007$); OC *vs* CR: $r = 0.43$ ($P = 0.08$)

**Figure 2** Relationship between changes in standardised mortality ratios (SMR) for oesophageal cancer in European countries, 1961–65 to 1971–75 and changes in per capita alcohol consumption, 1960 to 1960. **a**, male; **b**, female.

### Discussion

One concern of our methods relates to the use of standardised mortality ratios for cirrhosis to indicate the effect of alcohol consumption. Clearly not all chronic liver diseases and cirrhosis were alcohol-related. Donnan & Haskey (1977) had raised concerns about such use. However, Terris (1967) and Chilvers and her colleagues (1979) had found the index a reasonable one. In this study, we have not relied solely on mortality from cirrhosis but have in addition used per capia consumption figures as indicators of the effect of alcohol. It should be noted that such use has also been questioned in view of the apparent threshold effect of alcohol at population level shown by Møller *et al.* (1990). However, Tuyns & Audigier (1976), McMichael (1978) and Chilvers *et al.*, (1979) were able to demonstrate very clearly the effects of changes in mean consumption and trends in oesophageal cancer mortality in France, Australia and England and Wales respectively. Furthermore, in Australia and England and Wales, increases in oesophageal cancer mortality were found to be associated with increasing alcohol consumption at levels below 8 litres of ethanol per capita per year, which was the threshold level suggested by Møller and his colleagues (1990). In the same connection, one conceivable advantage in using mortality from cirrhosis as well is that deaths from cirrhosis may reflect more closely the prevalence of heavy users in a population.

Findings from case control studies in France (Tuyns *et al.*, 1977) and Italy (La Vecchia *et al.*, 1986b) showed that in men, about 90% of the risk of oesophageal cancer can be attributed to smoking and drinking. One could speculate that this would also apply in other European countries. The situation in women seems much less clear. From the present analysis of the time trend of standardised mortality ratios from oesophageal cancer between 1951 and 1985, it is evident that the changes followed a remarkably different pattern from those of lung cancer and cirrhosis, and per capita alcohol consumption figures. Despite increases in the latter three in most countries during the period studied, oesophageal cancer had either decreased or increased only slightly in most countries. Larger falls or only small rises in oesophageal cancer were seen in countries which had experienced smaller increases in lung cancer or cirrhosis. One problem with standardised mortality ratios relates to the dominance of these ratios by rates in the elderly so that their use may have obscured the trends in younger age groups. Indeed, Møller *et al.* (1990) recently reported that in many European male populations, oesophageal cancer had decreased among successive birth cohorts born before 1910 and had increased among those born after. This finding implies that in recent decades rates had decreased among the old and increased in the young.

In view of the problem with standardised morality ratios, we have also studied changes in age-specific rates. A similar pattern was found except for the youngest age group in men (50–59 years). The changes in younger men had been observed by Møller *et al.* (1990) and was attributed to increases in alcohol consumption. In many countries, oesophageal cancer mortality had decreased while lung cancer and cirrhosis had increased. The decrease in oesophageal cancer mortality was unlikely to be due to an improvement in treatment which remains difficult and survival is poor (Office of Population Censuses & Surveys, 1986). It is likely therefore that there has been a genuine decline in incidence rates of the conditon. This is supported by the evidence provided by incidence data available in some of the countries since the 1960s (Doll *et al.*, 1966; Doll *et al.*, 1970; Waterhouse *et al.*, 1976, 1982; Muir *et al.*, 1987). For women in the oldest group, the falls in oesophageal cancer mortality were accompanied by decreases in cirrhosis in many countries as well. Otherwise, to explain the decreasing trend, it is clear that we need to have a change in a risk/protective factor other than smoking and drinking.

Comparisons of the changes in standardised mortality ratios from oesophageal cancer in the two sexes revealed that they were closely correlated ($r = 0.85$), whereas those for lung cancer and cirrhosis were not. Correlation between changes in age-specific rates was also found in the 70–79 yeas age group ($r = 0.82$) and to a less extent the 60–69 years group ($r = 0.50$). The absence of correlation in lung cancer and cirrhosis was not surprising since the timing and magnitude of changes in consumption of alcohol and tobacco often differ between men and women in a population. Similarly, the increase in oesophageal cancer among younger men due to alcohol consumption was not accompanied by a similar change in women and this resulted in a lack of correlation ($r = -0.11$) between the degree of changes of the sexes in this age group. Such findings mean that the determining factor (or factors) of the decreasing trend of oesophageal cancer had acted more or less uniformly and simultaneously in men and women in individual populations. Dietary factors would probably act in such a way. In many populations, nutritional inadequacy has been found to be an important risk factor. Certain dietary changes in the populations may therefore have contributed to the decline seen for oesophageal cancer, as it has been proposed in parts of Scandinavia in relation to Plummer–Vinson syndrome (Muñoz & Day, 1992).

One possibility is the increased level of intake of fresh fruit and vegetable which had been shown to be protective in at least eight case control studies as reviewed by Muñoz and Day (1992). An examination of the temporal changes between 1948 and 1968 in the number of calories derived from different food categories in European countries using data compiled by the Food and Agriculture Organisation showed an increasing trend in fruit consumption in all countries (Food & Agriculture Organisation, 1971). However, the degrees of increase in the UK and Spain, where oesophageal cancer had increased, were among the lowest (data not shown). A negative correlation of changes in fruit consumption from 1948 to 1968 with changes in oesophageal cancer from 1956–60 to 1985 was found both in men ($r = -0.60$, $P < 0.05$) and in women ($r = -0.65$, $P < 0.05$). There was no association with the changes in consumption of vegetables or other categories (Table III). Although the correlation was not very high, it was probably as high as one could expect given the large number of possible items under the broad heading of 'fruits'. Furthermore, since the dietary measures available are clearly crude surrogate measures for dietary constituents of specific relevance, it is not meaningful to attempt to quantify the extent to which fruit consumption as recorded might explain the time trends of oesophageal cancer. The trends however are in the right direction, which is consistent with the suggestion that some fruit-related dietary components might have played an increasing and protective role over the past three to four decades. This finding and the results from case control studies indicate that fruit consumption may be a factor behind the differences in oesophageal cancer trend between European countries.

The findings of this study demonstrate that even with an attributable risk of 90% for alcohol and tobacco, other factors can have great potential for public health intervention. (The fact that the sum of attributable risks can exceed 100%, particularly for multiplicative interaction, is well known (Breslow & Day, 1980).) Their effects on the vulnerability of individual populations may also be modified by alcohol and tobacco. For example, the positive correlation between changes in age-specific rates of oesophageal cancer and lung cancer in the two older age groups among women (Tables IId and f) indicate that the decline of oesophageal cancer mortality brought about by changes in the undetermined factors was offset to a degree proportional to the carcinogenic load due to tobacco as reflected by lung cancer mortality. The results also imply that without the influence of alcohol and tobacco, oesophageal cancer could have decreased dramatically across Europe under the protective effect brought about by the change in the undetermined factor(s). Indeed, very large declines in oesophageal cancer have been observed in other populations, e.g. Singapore (Lee *et al.* 1988) and the Netherlands Antilles (Freni, 1984) and nutritional improvement had been put forward as an explanation. In this sense, an enormous opportunity might have been missed in Europe.

In conclusion, the present study shows that except in younger men, population-wide changes in certain undetermined risk/protective factor(s), one possibility of which is the consumption of fruit, may have overridden the effect of

**Table III** Correlation between changes in standardised mortality ratios for oesophageal cancer, 1956–60 to 1981–85 and changes in the number of calories derived from different food categories, 1948 to 1968

| | *Correlation coefficients* *Changes in standardised mortality ratios* | |
|---|---|---|
| | *Men* | *Women* |
| Changes in consumption of: | | |
| Cereals | 0.29 | 0.18 |
| Potatoes, starchy and other staple foods | 0.42 | 0.20 |
| Sugars and sweets | 0.25 | 0.07 |
| Pulses, nuts and seeds | − 0.07 | 0.20 |
| Vegetables | 0.05 | − 0.23 |
| Fruits | − 0.60* | − 0.65* |
| Meat | 0.10 | 0.10 |
| Eggs | − 0.18 | − 0.28 |
| Fish | − 0.29 | − 0.30 |
| Milk | 0.22 | 0.14 |
| Fats and oil | − 0.20 | − 0.35 |
| Total calorie | 0.23 | 0.13 |

*$P < 0.05$.

tobacco and alcohol and resulted in a reduction of oesophageal cancer risk. Apart from further efforts to reduce smoking and drinking, studies to identify the factor(s) will be of great public health importance in the prevention of oesophageal cancer.

We thank the World Health Organisation for providing the mortality statistics used in this study.

K.K.C. was on a British Commonwealth Scholarship. He also wishes to acknowledge the support from the Mary Sun Medical Scholarship Fund and the Cambridge-Hong Kong Link.

### References

BRESLOW, N.E. & DAY, N.E. (1980). *Statistical Methods in Cancer Research. Vol I. The Analysis of Case-Control Studies*. IARC Sci Publ 32. IARC: Lyon.

BROWN, M.M. & WALLACE, P. (1980). *Alcohol Beverage Taxation and Control Policies*. Brewers Association of Canada: Ottawa.

CHILVERS, C., FRASER, P. & BERAL, V. (1979). Alcohol and oesophageal cancer: an assessment of the evidence from routinely collected data. *J. Epidemiol. Community Health*, **33**, 127.

DOLL, R., PAYNE, P. & WATERHOUSE, J. (1966). *Cancer Incidence in Five Continents*. Springer Verlag: Berlin.

DOLL, R., MUIR, C. & WATERHOUSE, J. (1970). *Cancer Incidence in Five Continents*. IUCC: Geneva.

DONNAN, S. & HASKEY, J. (1977). Alcoholism and cirrhosis of liver. *Pop. Trends*, **7**, 18.

FOOD AND AGRICULTURE ORGANISATION (1971). *Production Yearbook 1970, Vol 24*. pp. 442–443. FAO: Rome.

FRENI, S.C. (1984). Long-term trends in the incidence rates of upper digestive tract cancer in the Netherlands Antilles. *Cancer*, **53**, 1618.

JENSEN, O.M., ESTEVE, J., MØLLER, H. & RENARD, H.J (1990). Cancer in the European Community and its member states. *Eur. J. Cancer*, **26**, 1167.

LA VECCHIA, C., DECARLI, A., MESSANOTTE, G. & CISLAGHI, C. (1986*a*). Mortality from alcohol realted disease in Italy. *J. Epidemiol. Community Health*, **40**, 257.

LA VECCHIA, C., LIATI, P., DECARLI, A., NEGRELLO, I. & FRANCESCHI, S. (1986*b*). Tar yield of cigarettes and the risk of oesophageal cancer. *Int. J. Cancer*, **38**, 381.

LEE, H.P., DUFFY, S.W., DAY, N.E. & SHANMUGARATNAM, K. (1988). Recent trends in cancer incidence among Singapore Chinese. *Int. J. Cancer*, **42**, 159.

MCMICHAEL, A.J. (1978). Increases in laryngeal cancer in Britain and Australia in relation to alcohol and tobacco consumption trends. *Lancet*, **i**, 1244.

MØLLER, H., BOYLE, P., MAISONNEUVE, P., LA VECCHIA, C. & JENSEN, O.M. (1990). Changing mortality from esophageal cancer in males in Denmark and other European countries, in relation to changing levels of alcohol consumption. *Cancer Causes and Control*, **1**, 181.

MUIR, C., WATERHOUSE, J., MACK, T., POWELL, J. & WHELAN, S. (1987). *Cancer Incidence in Five Continents, Vol V*. IARC: Lyon.

MUÑOZ, N., DAY, N.E. (1992). Esophagus. In *Cancer Epidemiology and Prevention*, 2nd ed. Schottenfeld, D., Fraumeni, J.F. Jr (eds). WB Saunders: New York (in press).

OFFICE OF POPULATION CENSUSES AND SURVEYS (1986). *Cancer Survival 1979–81 Registrations*. Office of Population Censuses & Surveys: London. (OPCS Monitor; Ref MB1 86/2.)

PARKIN, D.M., ÄÄRÄ, E. & MUIR, C.S. (1988). Estimates of the world-wide frequency of sixteen major cancers in 1980. *Int. J. Cancer*, **41**, 184.

TERRIS, M. (1967). Epidemiology of cirrhosis of the liver: national mortality data. *Am. J. Pub. Hlth.*, **57**, 2076.

TUYNS, A.J. & AUDIGIER, J.C. (1976). Double cohort increases for oesophageal and laryngeal cancer in France in relation to reduced alcohol consumption during the Second World War. *Digestion*, **14**, 197.

TUYNS, A.J., PÉQUIGNOT, G. & JENSEN, O.M. (1977). Le cancer de l'oesophage en Ille-et-Villaine en fonction des niveaux de consommation d'alcool et de tabac. Des risques qui se multiplient. *Bull. Cancer*, **64**, 45.

WATERHOUSE, J., MUIR, C., CORREA, P. & POWELL, J. (1976). *Cancer Incidence in Five Continents, Vol III*. IARC: Lyon.

WATERHOUSE, J., MUIR, C., SHANMUGARATNAM, K. & POWELL, J. (1982). *Cancer Incidence in Five Continents, Vol IV*. IARC: Lyon.